# 5′-tRF-GlyGCC: a tRNA-derived small RNA as a novel biomarker for colorectal cancer diagnosis

**DOI:** 10.1186/s13073-021-00833-x

**Published:** 2021-02-09

**Authors:** Yingmin Wu, Xiangling Yang, Guanmin Jiang, Haisheng Zhang, Lichen Ge, Feng Chen, Jiexin Li, Huanliang Liu, Hongsheng Wang

**Affiliations:** 1 Guangdong Key Laboratory of Chiral Molecule and Drug Discovery, School of Pharmaceutical Sciences, Sun Yat-sen University, Guangzhou, 510006 Guangdong China; 2 Guangdong Provincial Key Laboratory of Colorectal and Pelvic Floor Diseases, Guangdong Institute of Gastroenterology, The Sixth Affiliated Hospital, Sun Yat-sen University, Guangzhou, 510655 Guangdong China; 3 Department of Clinical Laboratory, The Sixth Affiliated Hospital, Sun Yat-sen University, Guangzhou, 510655 Guangdong China; 4 Department of Clinical Laboratory, The Fifth Affiliated Hospital, Sun Yat-sen University, Zhuhai, 519000 Guangdong China; 5 Department of Clinical Laboratory, Jinling Hospital, Medical School of Nanjing University, Nanjing, 210002 China

**Keywords:** *5′-tRF-GlyGCC*, CRC, ALKBH3, Plasma, tRNA-derived small RNAs

## Abstract

**Background:**

tRNA-derived small RNAs (tDRs), which are widely distributed in human tissues including blood and urine, play an important role in the progression of cancer. However, the expression of tDRs in colorectal cancer (CRC) plasma and their potential diagnostic values have not been systematically explored.

**Methods:**

The expression profiles of tDRs in plasma of CRC and health controls (HCs) are investigated by small RNA sequencing. The level and diagnostic value of 5′-tRF-GlyGCC are evaluated by quantitative PCR in plasma samples from 105 CRC patients and 90 HCs. The mechanisms responsible for biogenesis of 5′-tRF-GlyGCC are checked by in vitro and in vivo models.

**Results:**

*5′-tRF-GlyGCC* is dramatically increased in plasma of CRC patients compared to that of HCs. The area under curve (AUC) for 5′-tRF-GlyGCC in CRC group is 0.882. The combination of carcinoembryonic antigen (CEA) and carbohydrate antigen 199 (CA199) with 5′-tRF-GlyGCC improves the AUC to 0.926. Consistently, the expression levels of 5′-tRF-GlyGCC in CRC cells and xenograft tissues are significantly greater than that in their corresponding controls. Blood cells co-cultured with CRC cells or mice xenografted with CRC tumors show increased levels of 5′-tRF-GlyGCC. In addition, we find that the increased expression of 5′-tRF-GlyGCC is dependent on the upregulation of AlkB homolog 3 (ALKBH3), a tRNA demethylase which can promote tRNA cleaving to generate tDRs.

**Conclusions:**

The level of 5′-tRF-GlyGCC in plasma is a promising diagnostic biomarker for CRC diagnosis.

**Supplementary Information:**

The online version contains supplementary material available at 10.1186/s13073-021-00833-x.

## Background

Colorectal cancer (CRC) is one of the most common cancers worldwide and the fourth leading cause of cancer-induced death [30]. The survival of CRC patients is mostly affected by the stage of disease at the time of diagnosis. The 5-year survival rate of patients diagnosed at the localized stage is reported to be 90%, while it is diagnosed to be 10% at the regional or distant stage [24]. Thus, early screening is quite necessary for improving the clinical outcome of patients. Currently, the early screening and diagnosis of CRC are mainly based on fiber-optic colonoscopy and fecal occult blood (OB) testing such as carcinoembryonic antigen (CEA) and carbohydrate antigen 199 (CA199) [6, 21]. Colonoscopy is invasive, painful, expensive, and unsafe for patients [23]. Due to insufficient sensitivity and organ specificity, the OB test mainly used to detect the recurrence of CRC [6]. Therefore, identifying a simple, non-invasive, and high-diagnostic efficacy biomarker is urgently needed for early CRC screening and diagnosis.

tRNA-derived small RNAs (tDRs) are fragments of precursor or mature tRNAs that are usually 14~50 nucleotides (nt) in length [17]. According to the location of biogenesis, tDRs can be generally grouped into tRNA halves and tRNA-derived small RNA fragments (tRFs) [31, 37]. Further, tRFs can be further classified into 3 sub-groups: 5′-tRF, 3′-tRF, and inter tRF (i-tRF) [16, 18]. They are functionally diverse and associated with the regulation of gene expression, RNA processing, ribosome biogenesis, and LTR-retrotransposons [15, 26, 42]. The dysregulation of tDRs is associated with various human diseases, such as cancer, virus infection, metabolic disorder, and neurodegenerative diseases [3, 15, 42]. tDRs are associated with cancer progression via increasing cell proliferation in breast and prostate cancers [11]. Our recent study indicated that tRNA demethylase AlkB Homolog 3 (ALKBH3) can promote cancer progression via induction of tDRs [4].

The tDRs have been detected in body fluids such as blood and urine since the 1970s [1, 35]. Increasing evidence confirms the presence of high-abundant tDRs in different types of human cell lines, tissues, or extracellular body fluids [8, 11, 28, 33]. Recently, tDRs have been reported to distinguish between pre-from and post-seizure patients [10]. Consistently, emerging evidences indicated that tDRs might be used as potential biomarkers for certain types of cancer monitoring. For example, tDRs were dramatically increased in plasma exosomes of liver cancer patients, and four tDRs have the potential to become novel diagnostic biomarkers [41]. Circulating tDR-7816 expression is a novel potential biomarker for the diagnosis of patients with early non-triple-negative breast cancer [13].

In the present study, we investigated the expression profile of tDRs in plasma of CRC patients. The results showed that the level of *5′-tRF-GlyGCC* is higher in CRC patients than in healthy controls (HCs) and highlighted that *5′-tRF-GlyGCC* is a promising diagnostic biomarker for CRC patients. Its expression is positively correlated with ALKBH3 both in vitro and in vivo, respectively. Our results not only expand the distribution of non-coding RNAs in plasma, but also highlight the potential of tDRs as a promising biomarker for CRC diagnosis.

## Methods

### Human sample collection

Samples from 105 CRC patients and 95 HCs who had no history of basic or chronic diseases were collected from the Department of Clinical Laboratory of the Sixth Affiliated Hospital, Sun Yat-sen University, using tube for ethylene diamine tetraacetic acid (EDTA) anticoagulation. Unless otherwise stated, blood samples were centrifuged within 1 h of collection for 10 min at 3500 rpm at room temperature in a swing bucket centrifuge. Plasma samples were centrifuged at 13,000 r/min at 4 °C before the experiment. Separated plasma was transferred into a 1.5 mL RNase free polypropylene (PP) tube and stored at − 80 °C until RNA isolation within three months. In addition, 3 CRC patients and 3 HC samples with the detail information provided in Table S1 were collected for small RNA sequencing. All CRC patients were diagnosed on the basis of histopathology by biopsy or endoscopic examination. Plasma samples were collected at the time of diagnosis without surgery, chemotherapy, radiation, or any other kinds of treatment.

In order to evaluate the correlation between the expression of 5′-tRFGlyGCC in CRC tissues vs plasma of the same patients, 16-paired fresh tumor tissue and plasma were obtained from patients who underwent curative resection at the Sixth Affiliated Hospital, Sun Yat-sen University. None of the patients had received anticancer therapy before the sampling. Further, individuals with concurrent autoimmune disease, HIV, or syphilis were excluded.

The CRC stage was assessed by the TNM system according to the American Joint Committee on Cancer Staging Manual, Seventh Edition. Written informed consent was obtained from the patients. Ethic approval was obtained from the Ethics Committee of the Sixth Affiliated Hospital of Sun Yat-sen University (No. 2018ZSLYEC-008). Details of all samples are provided in Table S2.

### Small RNA library preparation and sequencing

The small RNA library preparation and sequencing were conducted according to our previous study [4]. Briefly, total RNA from plasma of 3 CRC patients and 3 HCs were extracted with Trizol reagent (Invitrogen, Carlsbad, CA, USA). Then, total RNAs were denatured and separated by a 15% TBE-Urea gel with 10/60 oligo length standard ladder (Integrated DNA Technologies, Coralville, IA). RNAs of 10 to 50 nt in gels were cut and recovered by small RNA PAGE recovery kit (Zymo Research, Irvine, CA, USA). After being treated with Tris for deacylation [8, 27] and T4 PNK for RNA end repair, the recovered tDRs were used for library preparation by use of NEB small RNA library preparation kit (E7330S). The purified libraries were quantified and validated. All 6 libraries were sequenced on an Illumina HiSeq 2500 (Illumina, San Diego, CA, USA).

### Quantitative real-time PCR

Total RNA from cell was isolated using TRIzol (Takara). The yield and purity of RNA were measured by NanoDrop 2000 (Thermo Fisher). cDNA was generated using the PrimeScript™ RT Master Mix (Takara) and small RNA first-strand was generated using the Mir-X™ miRNA First Strand synthesis kit (Takara). Real-time PCR was performed according to the protocol used in our previous study [4]. The expression of targeted genes and tRFs were normalized to GAPDH or U6, respectively. Primers of targeted genes were as follows: human *GAPDH*, forward 5′-GTC TCC TCT GAC TTC AAC AGC G-3′ and reverse 5′-ACC ACC CTG TTG CTG TAG CCA A-3′; mouse *GAPDH*, forward 5′-AGG TCG GTG TGA ACG GAT TTG-3′ and reverse 5′-TGT AGA CCA TGT AGT TGA GGT CA-3′; human *Alkbh3*, forward 5′-CCA CTG CTA AGA GCC ATC TCC A-3′ and reverse 5′-TCA ATC ACT CGT GGC TCA GGA G-3′; mouse *Alkbh3*, forward 5′-AGC CGC ATT GAA GAG AAC ACC AG-3′ and reverse 5′-CAT CGT CGC TGT GCC AGT CC-3′. *5′-tRF-GlyGCC*, forward 5′-GGC AGG CGA GAA TTC TAC CAC TGA ACC ACC AA-3′; *5′-half-GlyGCC*, forward 5′-GCA TGG GTG GTT CAG TGG TAG AAT TCT-3′; *5′-half-GluTTC*, forward 5′-TCC CAC ATG GTC TAG CGG TTA GG-3′; *5′-half-LysCTT*, forward 5′-GCC CGG CTA GCT CAG TCG-3′; *i-tRF-ArgCCT*, forward 5′-AGG GAT TGT GGG TTC GAG TCC-3′; and the reverse primer of all the small RNA used the mRQ 3′ universal primer.

### The quantification of *5′-tRF-GlyGCC*

To quantify the absolute amount of *5′-tRF-GlyGCC* in plasma, we established the standard curve for *5′-tRF-GlyGCC* by use of the synthesized standard (Sangon Biotech, Shanghai, China) with the sequence of 5′-GCA UGG GUG GUU CAG UGG UAG AAU UCU CGC C-3′. Setting up 8 concentration gradients of 40, 10, 1, 0.1, 0.01, 0.001, 0.0001, and 0.00001 ng respectively, the standard curve of y-coordinate was the △CT value for each of these concentrations measured by real-time PCR.

For each plasma sample, total RNA was isolated from 125 μl of plasma (human, BALB/c and BALB/c-nu-nu mice) using the TRIzol LS (Invitrogen) according to the manufacturer’s protocol. The small RNA first-strand was generated using the Mir-X™ miRNA First Strand synthesis kit (Takara) according to the manufacturer’s protocol in 1 μl RNA (10 μl RNA/125 μl plasma). The △CT value of each sample was measured by real-time PCR, and then the amounts of *5′-tRF-GlyGCC* were calculated based on the standard curve (shown in Fig. S1).

### Western blot analysis

Western blot analysis was performed as our previously described procedures [40]. The antibodies used in the present study were ALKBH3 (Millipore, BS1236, 1:3000) and GAPDH (BOSTER, BM3876, 1:1000).

### Cell line, cell culture, and transfection

Human CRC HCT116, SW620, HCT8, HCT15, SW480, RKO, CaCo2, HT29, Ls578T, cells, normal human colon epithelial cells NCM460, and human monocytic cell line THP-1 were obtained from American Type Culture Collection (Manassas, VA, USA) and maintained in our lab. Non-tumorigenic human peripheral blood B lymphocyte PENG-EBV cells were obtained from Kunming Cell Bank (Chinese Academy of Sciences). The mouse CRC cell CT26 was also purchased from the American Type Culture Collection (Manassas, VA, USA). All cells were maintained in our lab with cultured in RPMI 1640 or DMEM medium (GIBCO, Carlsbad, CA, USA) with 10% fetal bovine serum (FBS) and 1% 100 × Pen/Strep (Thermo Fisher) in a 5% CO_2_ cell culture incubator at 37 °C.

Cell co-culture system was used to evaluate the effects of CRC cells on the expression of *5′-tRF-GlyGCC*. Firstly, blood PENG-EBV or THP-1 cells were plated onto the bottom of a 6-well plate at the density of 2 × 10^5^ cells/well, and CRC cell lines (SW620, HCT116, RKO, SW480, HCT8, and HCT15) were seeded onto the inside of an insert Transwell chamber (polyethylene terephthalate hanging cell culture insert with pore size of 0.4 μm, Millipore) with a density of 2 × 10^5^ cells/well. Co-culture was started by setting the insert on the 6-well plate. To evaluate the roles of ALKBH3 in the expression of *5′-tRF-GlyGCC*, PENG-EBV and CRC cells (HCT8, HCT15, SW620, RKO, SW480) were transfected with ppB-*Alkbh3* or sh-*Alkbh3* and corresponding vector controls via liposome-mediated transfection.

### Animal studies

BALB/c-nu-nu mice and BALB/c mice (4 weeks old) were purchased from Sun Yat-sen University (Guangzhou, China) Animal Center and raised under pathogen-free conditions. All animal experiments complied with Zhongshan School of Medicine Policy on Care and Use of Laboratory Animals. To establish CRC xenograft model in mice, human CRC cell line HCT116 and mouse CRC cell line CT26 (5 × 10^6^ cells in 150 μl PBS + 150 μl Matrigel) were injected into right flanks of BALB/c-nu-nu mice (*n* = 6, male to female = 1:1) and BALB/c mice (*n* = 6, male to female = 1:1), respectively. PBS with 150 μl was also injected into right flanks of BALB/c-nu-nu mice (*n* = 6, male to female = 1:1) and BALB/c mice (*n* = 6, male to female = 1:1) as the control group, respectively. Mice were euthanized 30 days after cell/PBS injection or if the longest dimension of the tumors reached 20 mm before 30 days. Immediately following euthanasia, blood samples were collected from mice orbit using the tubes for EDTA anticoagulation. Then, 125 μl blood per sample was used to extract RNA and the remaining blood was further centrifugation to collect plasma.

### Statistical analysis

Statistical analysis was performed with IBM SPSS Statistics 15.0, GraphPad Prism version 7.0, and MedCalc version 17.1. Data were reported as mean ± standard deviation (SD) from at least three independent experiments unless otherwise specified. Data were analyzed by two-tailed unpaired Student’s *t* test between two groups and by one-way ANOVA followed by Bonferroni test for multiple comparisons. Spearman correlation test was used for correlation analysis. All statistical tests were two-sided. A *p* value of < 0.05 was considered to be statistically significant. *p<0.05, **p<0.01, ***p <0.001, ****p < 0.0001; ns, no significant.

## Results

### The expression profiles of tDRs in plasma of CRC and HCs

**Fig. 1:**
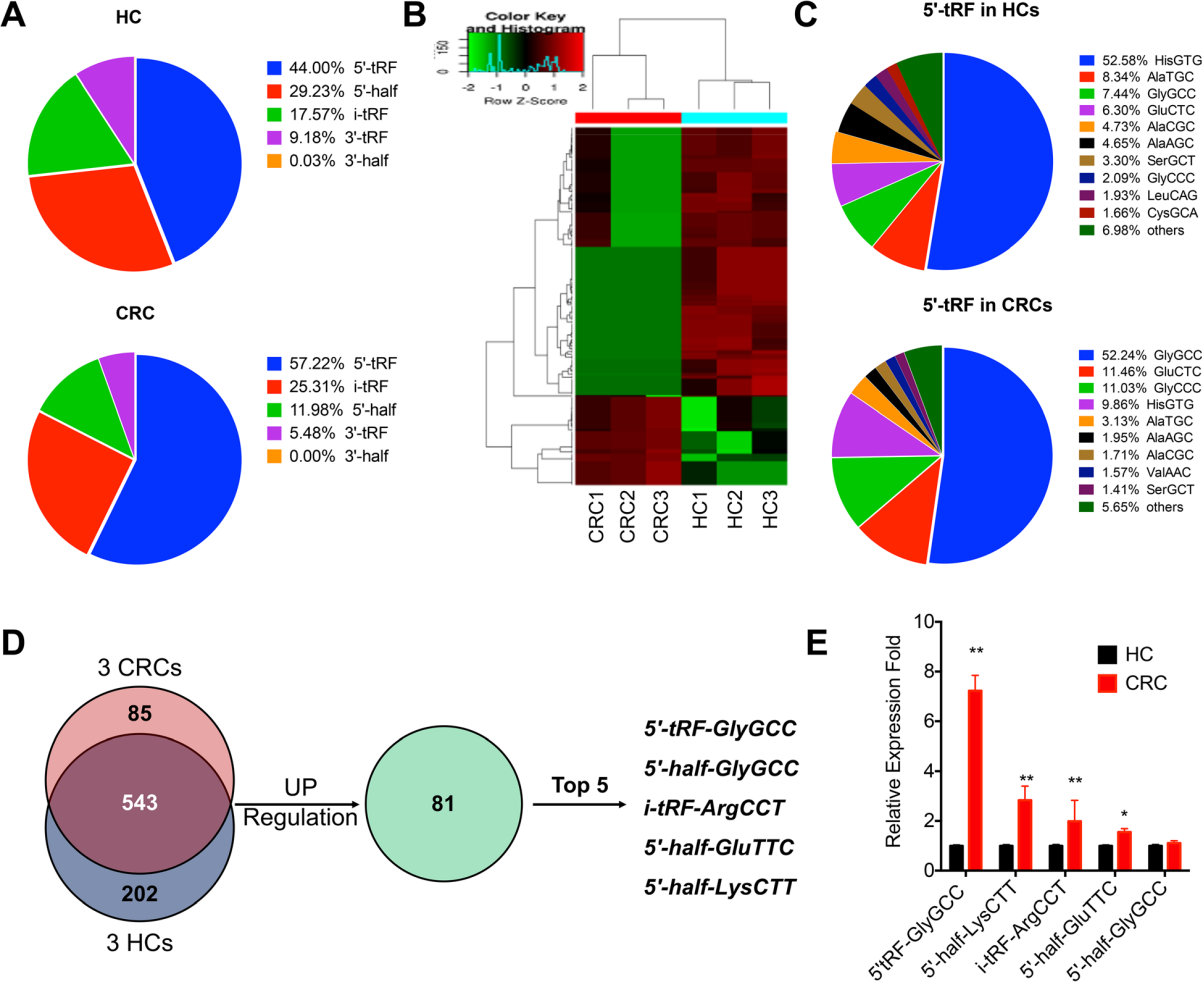
The expression profiles of tDRs in plasma of CRC and HCs. **a** Percentage of each type of tDRs in CRC and HCs plasma. **b** Heat maps of tRFs that were differentially expressed between 3 CRC plasma (*left*) and 3 HC plasma samples (*right*). The color scale shown on the upper left illustrates the relative tRF expression levels; red represents high expression, and green represents low expression. **c** The expression profiles of 5′-tRF in CRC and HCs plasma. **d** Venn diagram showing the overlapping of tDRs increased in 3 CRC plasma with the fold greater than 2 and adjusted *p* value less than 0.05. **e** The levels of 5 tDRs in plasma of 3 CRC patients and 3 healthy control were verified by qRT-PCR. Data are presented as the mean ± SD from three independent experiments. *p < 0.05, **p < 0.01 compared with control

To investigate the expression profiles of tDRs in plasma of CRC and HCs, small RNAs (smRNA) ranging from 10 to 50 bp from plasma samples of 3 CRC and 3 HC subjects (Table S1) were analyzed using small RNA high-throughput sequencing. The detailed reads data were listed in Table S3. Profiling assay indicated that the abundance of tDRs decreased with the order of 5′-tRF > 5′-half/i-tRF > 3′-tRF > 3′-half in HCs and CRC plasma (Fig. 1a). Further, the proportion of 5′-tRF in CRC plasma was significantly greater than that in HCs (Fig. 1a), suggesting that 5′-tRF might be involved in CRC tumorigenesis and progression. The expression of individual tDR profiles was further analyzed. Hierarchical clustering showed systematic variations in tDR expression in plasma between HCs and CRC (Fig. 1b), and 628 and 745 tDRs were obtained in CRC and HCs, respectively.

The differentially expressed 5′-tRFs in plasma between CRC and HCs were further analyzed. The results showed that the 5′-tRF profiles in CRC plasma were largely different from that in HC plasma (Fig. 1c). For example, the highest abundance 5′-tRF in plasma of HC and CRC was *5′-tRF-HisGTG* and *5′-tRF-GlyGCC*, respectively. The expression of *5′-tRF-GlyGCC*, which accounted for 7.44% of 5′-tRF in HC plasma, increased to 52.24% of 5′-tRF in CRC plasma. In addition, the proportions of *5′-tRF-GlyCCC* markedly increased in CRC plasma; however, the percentage of *5′-tRF-HisGTG* and *5′-tRF-AlaTGC* obviously decreased in CRC plasma (Fig. 1c). All these data suggested that the profiles of 5′-tRFs in CRC plasma were significantly different from that in HCs.

Among the 628 and 745 tDRs identified in CRC and HCs, there were 85 and 202 unique tDRs for each group, respectively (Fig. 1d). Further, 81 tDRs were significantly increased in plasma of CRC than that in HCs (Fig. 1d). To validate the findings of small RNA sequencing, the 5 upregulated candidate tDRs in the above same 3 CRC and 3 HC subjects plasma samples were further checked by qRT-PCR. As showed in Fig. 1e, all measured tDRs in CRC plasma increased compared to that of HCs, while the increase of *5′-tRF-GlyGCC* was the highest among the five measured ones. All these data revealed that expression profiles of tDRs were variated in plasma of CRC as compared with that in HCs; further, 5′-tRF, particularly *5′-tRF-GlyGCC*, were significantly increased in CRC plasma.

### The levels of *5′-tRF-GlyGCC* in plasma of CRC patients

**Fig. 2:**
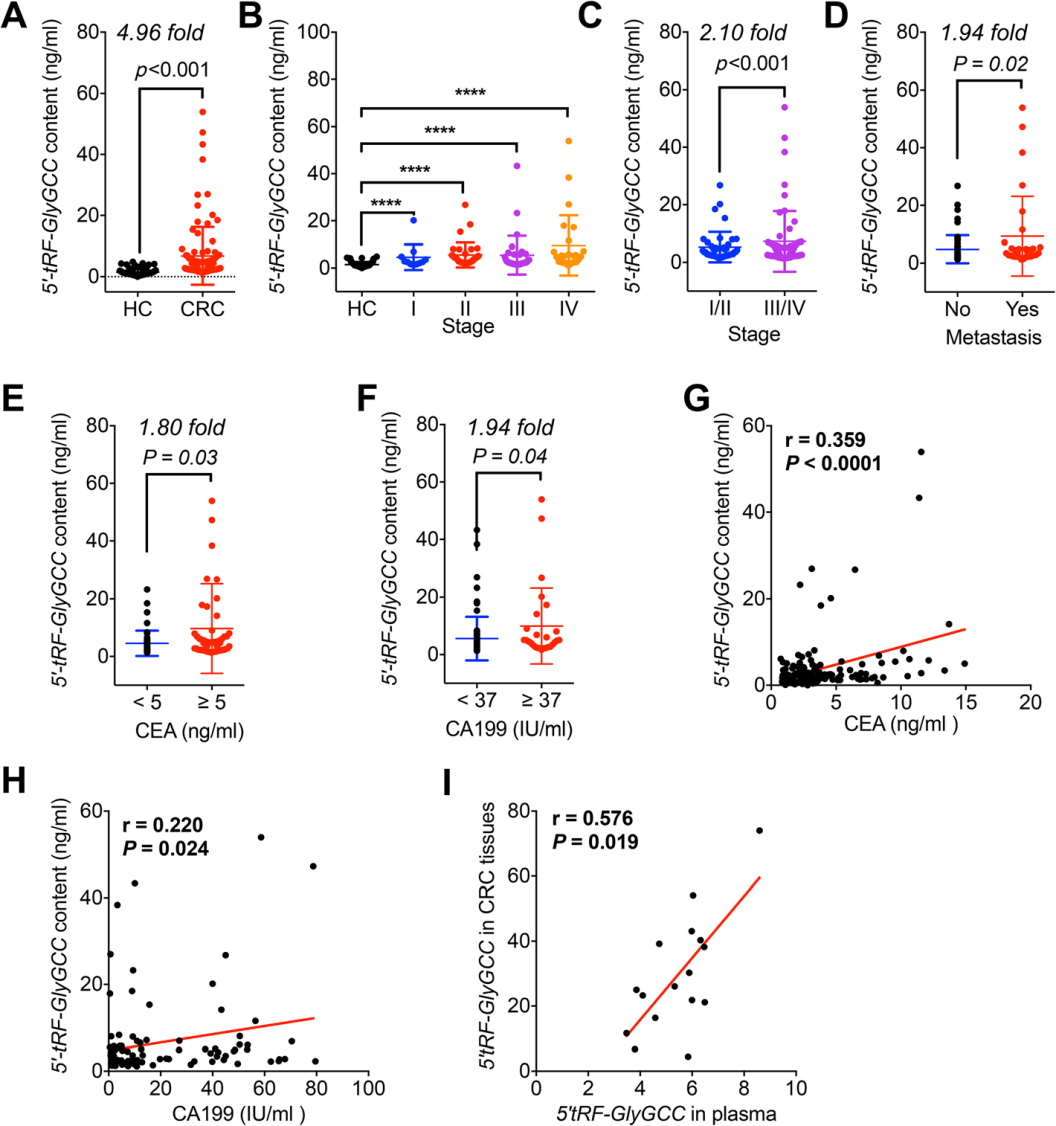
The levels of *5′-tRF-GlyGCC* in plasma of CRC patients. **a** The amount and relative fold of *5′-tRF-GlyGCC* in plasma of CRC patients (*n* = 105) and HC (*n* = 90). **b** The amount of *5′-tRF-GlyGCC* in plasma of different pathological stages of CRC patients (stage I, *n* = 11; stage II, *n* = 34; stage III, *n* = 32; stage IV, *n* = 25). **c** The comparison of *5′-tRF-GlyGCC* levels in plasma of stage I/II (*n* = 45) and III/IV (*n* = 57) CRC patients. **d** The comparison of *5′-tRF-GlyGCC* levels in plasma of CRC patients with (*n* = 30) or without (*n* = 64) metastasis. **e** The comparison of *5′-tRF-GlyGCC* levels in plasma of CRC patients with CEA ≥ 5 ng/ml (*n* = 54) or CEA < 5 ng/ml (*n* = 47). **f** The comparison of *5′-tRF-GlyGCC* levels in plasma of CRC patients with CA199 ≥ 37 IU/ml (*n* = 27) or CA199 < 37 IU/ml (*n* = 74). **g**, **h** The Spearman correlation of *5′-tRF-GlyGCC* amount with the levels of CEA (**g**) or CA199 (**h**) in CRC patients. **i** The Spearman correlation of *5′-tRF-GlyGCC* amount in paired plasma and tumor tissues from 16 CRC patients. *p < 0.05, **p < 0.01, ***p < 0.001, ****p < 0.0001

*5′-tRF-GlyGCC* located at chromosomes 1, 2, 6, 16, and 17 with a transcript length of 31 nt, and the sequence is 5′-GCA UGG GUG GUU CAG UGG UAG AAU UCU CGC C-3′ (MINTbase Unique ID: tRF-35-PNR8YP9LON4VN1). The small RNA-seq data suggested that the *5′-tRF-GlyGCC* increased in CRC patients was greater than that of other tDRs. Consistently, *5′-tRF-GlyGCC* was one of the most abundant tDRs detected in human cells and tissues in our previous study [4]. We therefore further checked its expression in plasma of patients with CRC (*n* = 105) and HCs (*n* = 90). Our data showed that the abundance of *5′-tRF-GlyGCC* was significantly higher in CRC patients than in HCs (*p* < 0.0001, Fig. 2a). We then analyzed the expression of *5′-tRF-GlyGCC* in different pathological stages of CRC patients (stage I, *n* = 11; stage II, *n* = 34; stage III, *n* = 32; stage IV, *n* = 25). The results showed that the amount of *5′-tRF-GlyGCC* at each stage of CRC was higher than of HCs (*p* < 0.0001, Fig. 2b). The results indicated that *5′-tRF-GlyGCC* might be a promising biomarker for the diagnosis of CRC patients.

We further evaluated the expression of *5′-tRF-GlyGCC* with the progression of CRC. The *5′-tRF-GlyGCC* expression was observed to increase as CRC progressed (Fig. 2b). In addition, the abundance of *5′-tRF-GlyGCC* in stage I/II CRC patients was significantly less than that in stage III/IV patients (Fig. 2c). Moreover, metastasized CRC patients had significantly higher levels of *5′-tRF-GlyGCC* in plasma than those of no metastasis group (*p* < 0.05, Fig. 2d). Also, CRC patients with CEA ≥ 5 ng/ml had significantly greater levels of *5′-tRF-GlyGCC* than that of patients with CEA < 5 ng/ml (Fig. 2e). Further, CRC patients with CA199 ≥ 37 IU/ml had significantly greater levels of *5′-tRF-GlyGCC* than that of CA199 < 37 IU/ml ones (Fig. 2f). In addition, results showed that the levels of *5′-tRF-GlyGCC* in plasma were significantly positively correlated with the levels of CEA (Fig. 2g) and CA199 (Fig. 2h) for CRC patients. However, the distribution of *5′-tRF-GlyGCC* in CRC patients seems to be independent of the statuses of gender, age, colon or rectal location, KRAS mutation, MSI/MSS, Ki67, CA125, CA15-3, or FAP expression (Summarized in the Table S4). The results showed that *5′-tRF-GlyGCC* was upregulated in plasma of CRC patients and increased with the progression and metastasis of CRC.

Further, we checked whether there was a correlation between the expression of *5′-tRF-GlyGCC* in CRC tissues and plasma of the same patients by collection of 16 paired tumor tissues and plasma samples. The results showed that the expression of *5′-tRF-GlyGCC* in plasma was significantly correlated with that in the paired CRC tissues (Fig. 2i). It indicated that patients with greater levels of *5′-tRF-GlyGCC* in plasma also had higher levels of *5′-tRF-GlyGCC* in CRC tissues.

### Diagnostic value of *5′-tRF-GlyGCC* as a biomarker for CRC patients

**Fig. 3:**
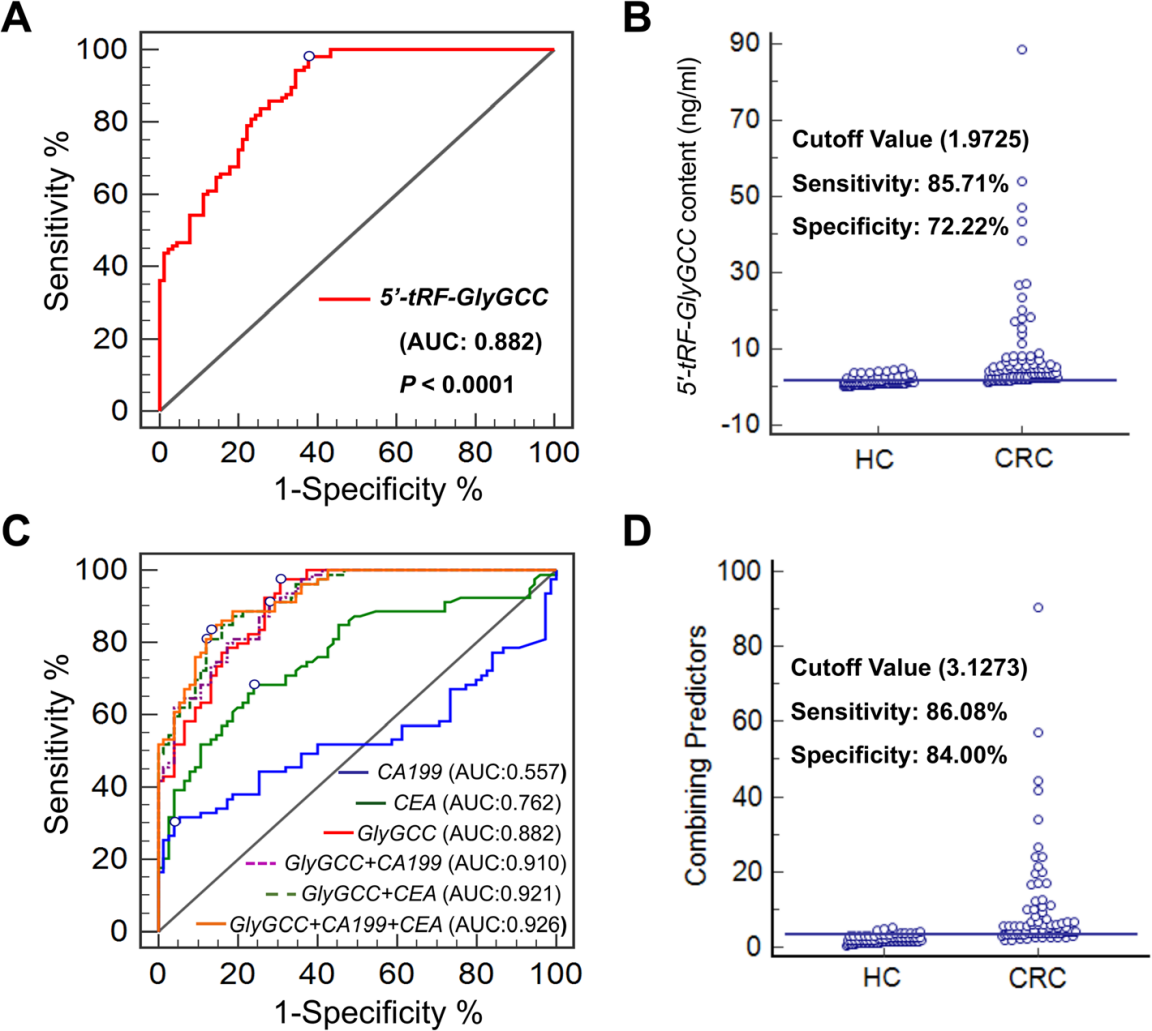
Diagnostic value of *5′-tRF-GlyGCC* as a biomarker for CRC patients. **a**, **b** The ROC curve (**a**) and cutoff value (**b**) for *5′-tRF-GlyGCC* including CRC patients and HC. **c**, **d** The ROC curves (**c**) for *5′-tRF-GlyGCC*, CEA, CA199 alone or together and cutoff value (**d**) for *5′-tRF-GlyGCC*, CEA, CA199 in combination including CRC patients and HC

To test whether plasma *5′-tRF-GlyGCC* levels had diagnostic value for CRC patients, a receiver-operating characteristic (ROC) curve was plotted to identify a cutoff value. As shown in Fig. 3a and Table S5, plasma *5′-tRF-GlyGCC* content could distinguish CRC patients from HCs, with an area under the curve (AUC) of 0.882 (95% CI 0.83 to 0.92, *p* < 0.0001). The optimal cutoff value for *5′-tRF-GlyGCC* was 1.9725 (sensitivity 86%, specificity 72%) (Fig. 3b). The results showed that the diagnosis value of *5′-tRF-GlyGCC* was much better than that of CEA (AUC 0.762) or CA199 (0.557). Further, the ROC curves of the combination of *5′-tRF-GlyGCC*, CEA, and CA199 improved the AUC value to 0.926 (95% CI 0.87–0.96, *p* < 0.0001) (Fig. 3c). The combining optimal cutoff value was 3.1273 (sensitivity 86%, specificity 84%) (Fig. 3d). This indicated that the plasma levels of *5′-tRF-GlyGCC* provided excellent diagnostic capabilities for CRC patients.

### ALKBH3 is involved in the biogenesis of *5′-tRF-GlyGCC*

**Fig. 4:**
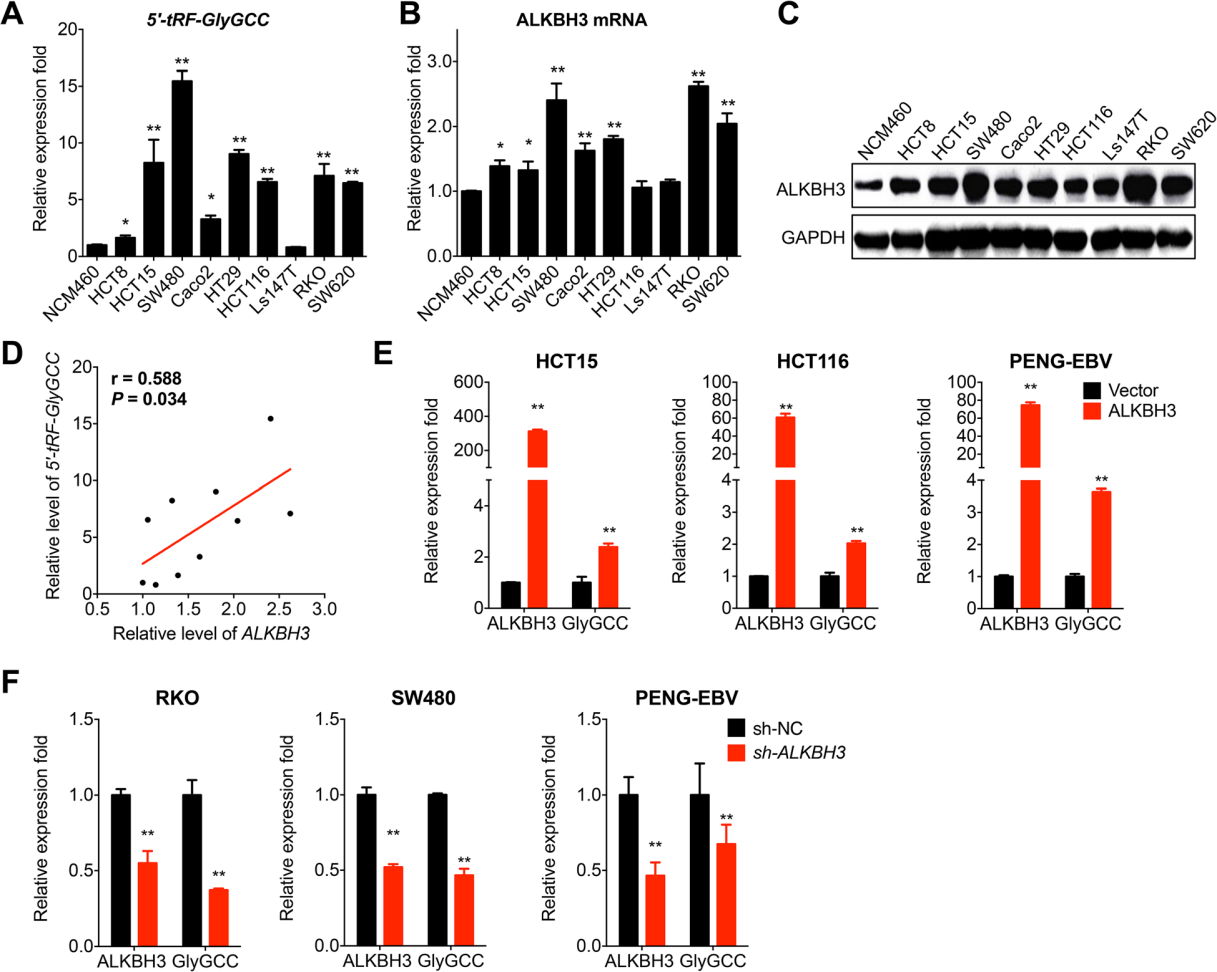
ALKBH3 is involved in the biogenesis of *5′-tRF-GlyGCC*. **a**, **b** The expression of 5′-tRF-GlyGCC (**a**) and mRNA of ALKBH3 (**b**) in CRC cell lines and human colon mucosal epithelial NCM460 cells were measured by qRT-PCR. **c** The protein expression of ALKBH3 in human CRC and NCM460 cells. **d** The Spearman correlation between the levels of 5′-tRF-GlyGCC and the mRNA expression of ALKBH3 in CRC and NCM460 cells. **e** Cells were transfected with the vector control or PPB/ALKBH3 for 48 h, and the expression of ALKBH3 and 5′-tRF-GlyGCC were measured by qRT-PCR. **f** Cells were transfected with the sh-NC control or sh-ALKBH3 for 48 h, and then the expression of ALKBH3 and 5′-tRF-GlyGCC were measured by qRT-PCR. Data are presented as the mean ± SD from three independent experiments. *p < 0.05, ** *p* < 0.01 compared with control

Our previous study reported that ALKBH3 was a tRNA demethylase and has only been identified as inducing the generation of tDRs [4]. We then investigated whether ALKBH3 was involved in the biogenesis of *5′-tRF-GlyGCC*. Firstly, the expression of *5′-tRF-GlyGCC* and ALKBH3 were measured in nine human CRC cell lines and human colon mucosal epithelial cells NCM460. The results showed that both *5′-tRF-GlyGCC* (Fig. 4a) and ALKBH3 mRNA (Fig. 4b) in most measured CRC cell lines was significantly greater than that in NCM460 cells. Consistently, western blot analysis confirmed that the protein expression of ALKBH3 was upregulated in CRC cells (Fig. 4c). Further, the expression of *5′-tRF-GlyGCC* was significantly and positively correlated with the mRNA levels of ALKBH3 in the measured CRC cells (Fig. 4d).

Further, we then overexpressed ALKBH3 in HCT15, HCT116 (ALKBH3 low CRC cells), and blood PENG-EBV cells via transient transfection (Fig. S2A). The results showed that over expression of ALKBH3 can increase the expression of *5′-tRF-GlyGCC* in all examined cells (Fig. 4 E). We knocked down the expression of ALKBH3 in CRC cell SW480, RKO (ALKBH3 high expression CRC cells), and PENG-EBV cells (Fig. S2B). The results showed that ALKBH3 silencing downregulated the level of *5′-tRF-GlyGCC* in SW480, RKO, and PENG-EBV cells (Fig. 4f). Consistently, overexpression of ALKBH3 in RKO and SW480 cells can increase the expression of *5′-tRF-GlyGCC* (Fig. S4 C), while knockdown the expression of ALKBH3 can decrease the expression of *5′-tRF-GlyGCC* in HCT15 and HCT116 cells (Fig. S4 D). Collectively, our data showed that the tRNA demethylase ALKBH3 was involved in the biogenesis of *5′-tRF-GlyGCC* in both CRC and blood cells.

### Co-culture with CRC cells increased *5′-tRF-GlyGCC* of blood cells via ALKBH3

**Fig. 5:**
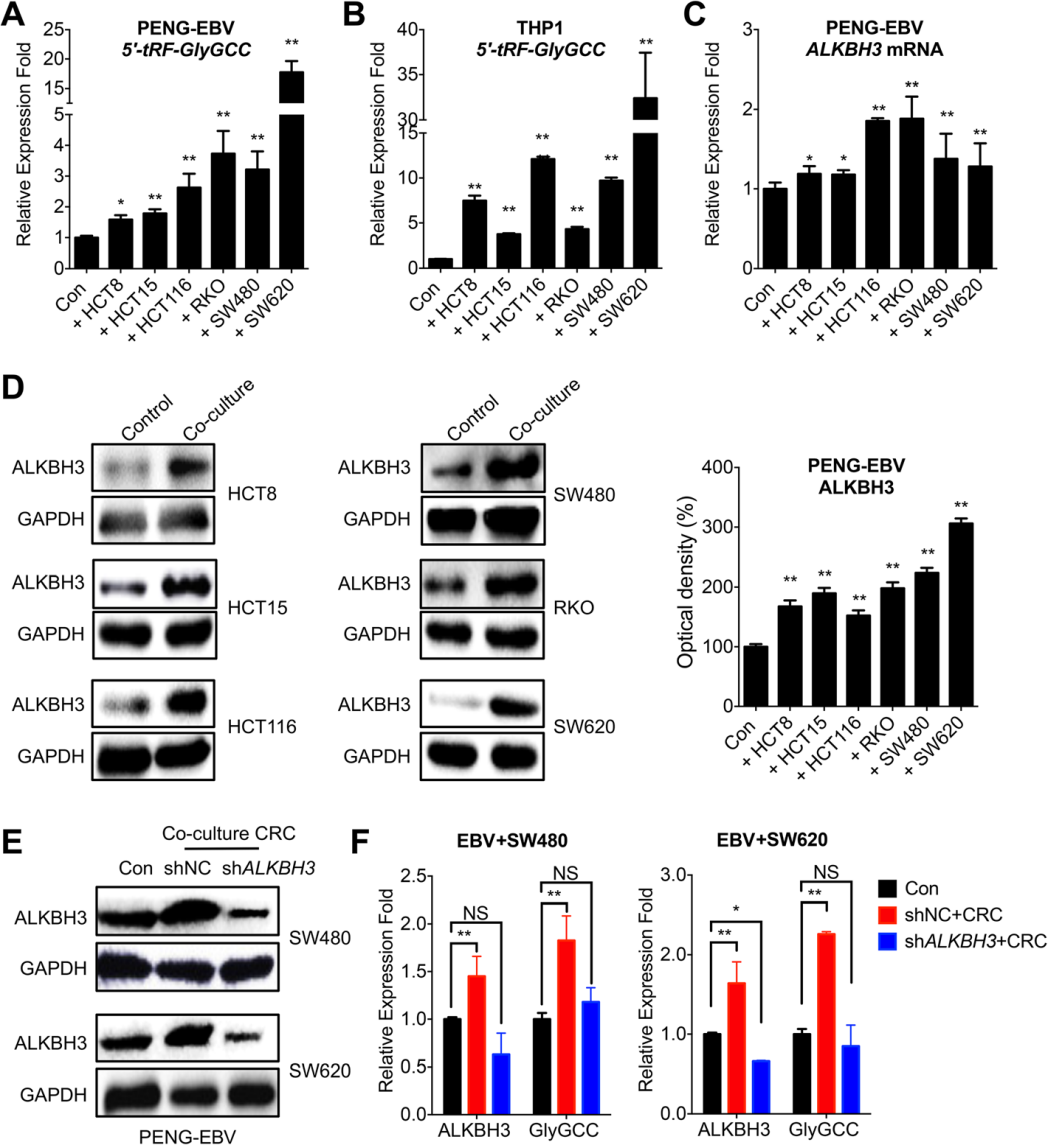
Co-culture with CRC cells increased *5′-tRF-GlyGCC* of blood cells via ALKBH3. **a**, **b** The levels of 5′-tRF-GlyGCC in PENG-EBV (**a**) or THP-1 (**b**) cells co-cultured with or without CRC cells was tested by RT-PCR. **c** The levels of ALKBH3 mRNA in PENG-EBV cells co-cultured with or without CRC cells were tested by RT-PCR. **d** The protein levels of ALKBH3 in PENG-EBV cells co-cultured with or without CRC cells were checked by western blot analysis and quantitatively analyzed. **e**, **f** PENG-EBV cells pre-transfected with sh-control or sh-ALKBH3 were further co-cultured with or without CRC cells for 48 h; the expression of ALKBH3 **e** and 5′-tRF-GlyGCC **f** in PENG-EBV cells were checked. Data are presented as the mean ± SD from three independent experiments. *p < 0.05, ** *p* < 0.01 compared with control; NS, no significant

We further investigated the potential mechanisms responsible for upregulation of *5′-tRF-GlyGCC* in plasma of CRC patients. Since circulating tumor cells are widespread in the peripheral blood of CRC patients [9, 25], we then used Transwell chamber (pore size of 0.45 μm) to establish a co-culture system of peripheral blood cells PENG-EBV and human monocytic cell THP-1 with CRC cells, respectively. Our data showed that co-culture with all examined CRC cells can significantly increase the *5′-tRF-GlyGCC* levels in PENG-EBV cells (Fig. 5a). Consistently, co-culture with CRC cells can significantly increase the *5′-tRF-GlyGCC* levels in THP-1 cells (Fig. 5b). In addition, when co-cultured with all CRC cells, the mRNA expression of ALKBH3 in PENG-EBV cells also increased significantly (Fig. 5c). Further, western blot analysis confirmed that co-culture with CRC cells increased the protein expression of ALKBH3 in PENG-EBV cells (Fig. 5d). The culture medium of CRC cells could also induce the expression of *5′-tRF-GlyGCC* in both PENG-EBV and THP-1 cells while the boiled medium (100 °C for 5 min) had no similar effect (data not shown), which suggested the CRC secreted cytokines or other substances can induce expression of *5′-tRF-GlyGCC.*

We further investigated whether ALKBH3 is involved in CRC-induced 5′-tRF-GlyGCC of blood cells. The expression of ALKBH3 was knocked down in PENG-EBV cells (Fig. 5e). The results showed that the deletion of ALKBH3 can attenuate expression of *5′-tRF-GlyGCC* in PENG-EBV cells induced by SW620 (Fig. 5f) and SW480 (Fig. 5g). It suggested that CRC cells can increase the *5′-tRF-GlyGCC* levels in peripheral blood cells via upregulation of ALKBH3.

### Xenografted CRC tumors increased levels of *5′-tRF-GlyGCC* in plasma of mice

**Fig. 6:**
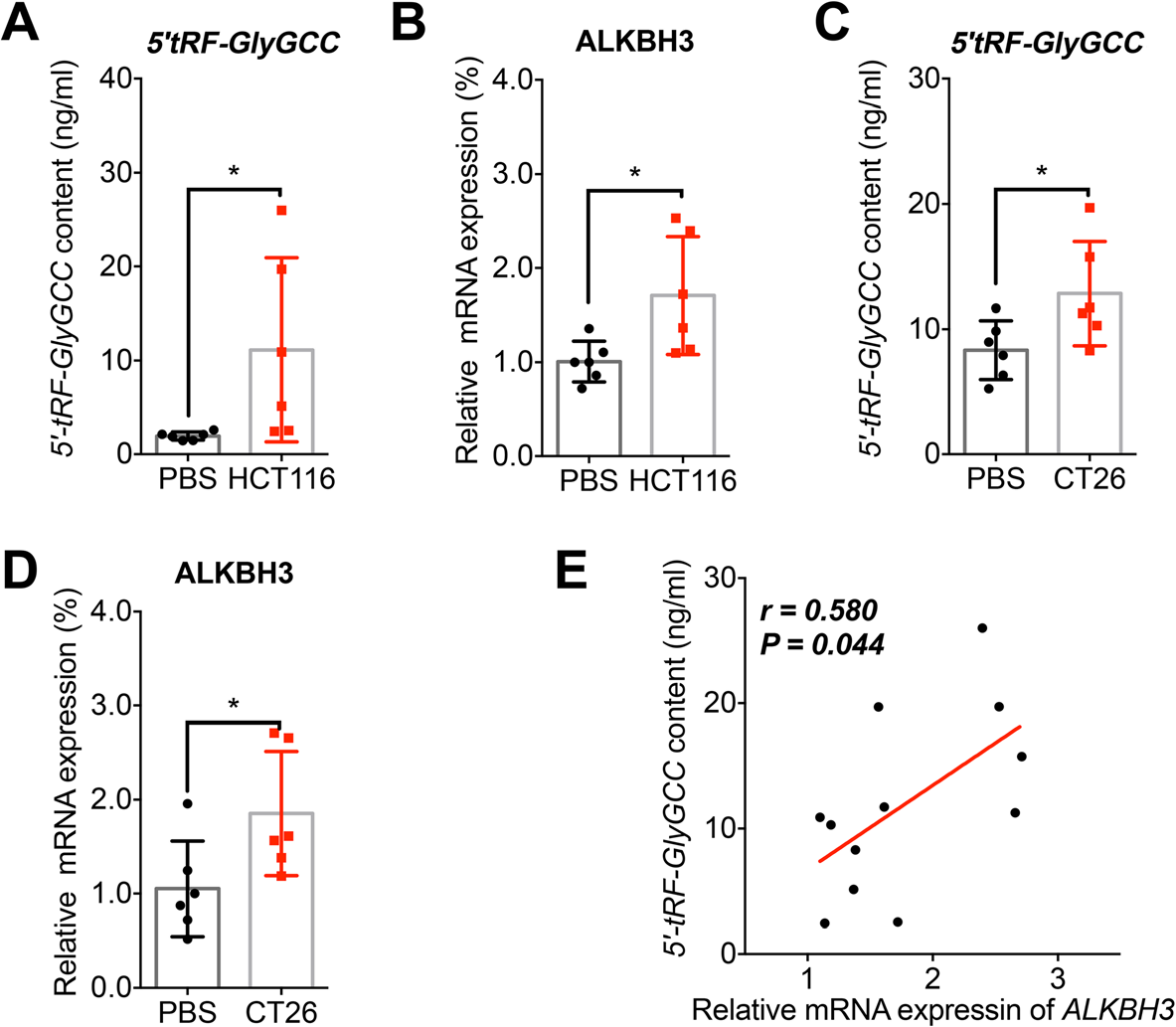
Xenografted CRC tumors increased levels of *5′-tRF-GlyGCC* in plasma of mice. **a** Levels of *5′-tRF-GlyGCC* in plasma of nude mice of control (*n* = 6) or bearing human CRC HCT116 tumor (*n* = 6). **b** Relative mRNA level of ALKBH3 in nude mice blood of control (*n* = 6) or bearing human CRC HCT116 tumor (*n* = 6). **c** Levels of *5′-tRF-GlyGCC* in plasma of BALB/c mice of control (*n* = 6) or bearing mouse CRC CT26 tumor (*n* = 6). **d** Relative mRNA level of ALKBH3 in BALB/c mice blood control (*n* = 6) or bearing mouse CRC CT26 tumor (*n* = 6). **e** The Spearman correlation between the levels of *5′-tRF-GlyGCC* and the mRNA expression of ALKBH3 in xenografted mice. *p < 0.05 as compared with the PBS group

To evaluate the in vivo effects of CRC tumors on *5′-tRF-GlyGCC* expression, we established the human CRC HCT116 xenografts by using of BALB/c-nu-nu mice (*n* = 6, male to female = 1:1, Fig. S3A). Our data showed that the plasma levels of *5′-tRF-GlyGCC* significantly increased in xenografted nude mice as compared to those in the control group (*p* < 0.05, Fig. 6a). Further, the mRNA expression of ALKBH3 in peripheral blood significantly increased (*p* < 0.01, Fig. 6b). Then, mouse CRC CT26 cells were used to establish xenografts with BALB/c mice (*n* = 6, male to female = 1:1, Fig. S3B). Consistently, the plasma levels of *5′-tRF-GlyGCC* (Fig. 6c) and the mRNA expression of ALKBH3 in peripheral blood (Fig. 6d) significantly (*p* < 0.05) increased in CT26 xenografted BALB/c mice. In addition, the expression of *5′-tRF-GlyGCC* level was significantly and positively associated with the expression of ALKBH3 in tumor-bearing mice (Fig. 6e). These results indicated that CRC xenograft can promote plasma levels of *5′-tRF-GlyGCC*, accompanied by the up-regulation of ALKBH3 in vivo*.*

## Discussion

The detection of precursor lesions and early-onset CRC is critical for prevention and therapy of this disease. Colonoscopy is considered as the gold standard for CRC screening; however, it is invasive, expensive, and has low compliance rates and complications such as hemorrhage and perforation [22]. While the most commonly used non-invasive screening tests such as fecal OB testing and fecal immunochemical test (FIT) have lower sensitivity and specificity [34], there is an imperative need to develop novel and robust non-invasive strategies for CRC diagnosis. Further, biomarkers that enable the proper selection of patients would be helpful for the improvement of therapy efficiency. In this study, we found that plasma level of *5′-tRF-GlyGCC* could be a novel biomarker to screen for CRC. In addition, the combination with the current tumor markers such as CEA and CA199 can further enhance its diagnostic values.

With the development of sequencing technology, emerging evidence revealed that dysregulation of tDRs was involved in the progression of human diseases, such as cancer, neurodegenerative diseases, and inherited metabolic disorders [8, 11, 28, 33]. Among the few tDRs that have been functionally characterized, tRF/miR-1280, a 17-nt fragment derived from tRNA^Leu^ and pre-miRNA, suppressed CRC growth and metastasis [12]. Enriched 5′-tiRNA-Val was observed in CRC patients and correlated with tumor metastasis [19]. Also, several recent studies have revealed that tDRs were non-invasive diagnostic biomarkers for various diseases including cancer [7, 10, 31, 43].

By small RNA sequencing, we identified that the profile of tDRs in plasma of CRC was significantly different from that of HCs. This is particularly true for the expression of 5′-tRF and *5′-tRF-GlyGCC*, whose levels significantly increased in CRC plasma. The AUC for *5′-tRF-GlyGCC* in CRC group was 0.882 (95% CI, 0.83–0.92), which is markedly greater than that of CEA and CA199. The combination of CEA and CA199 with 5′-tRF-GlyGCC improved the AUC to 0.926 (95% CI, 0.87–0.96). It has been revealed that *tRF-25*, *tRF-38*, and *tRF-18* can be used to diagnose osteoporosis with an average AUC of 0.815 [38]. As to CRC diagnosis, circulating microRNAs can be used as useful non-invasive diagnostic biomarkers with AUC values ranged from 0.6 to 0.9 [2]. However, so far, there is no single stand-alone miRNA that has yet been identified as an ideal biomarker for the diagnosis of CRC [14]. Our data showed for the first time that plasma levels of 5*′-tRF-GlyGCC* significantly increased in the CRC group as compared to those in the HC group. Further, blood cells co-cultured with CRC cells or mice xenografted with CRC tumors showed increased levels of 5*′-tRF-GlyGCC*. All these data suggested that 5*′-tRF-GlyGCC* might be a robust biomarker for CRC diagnosis. Considering that 5*′-tRF-GlyGCC* has been reported to be critical for the progression of other cancers such as breast [36] and lung [4] cancer, whether it will be used as a biomarker for other cancers requires further study.

We further found that the tRNA demethylase ALKBH3 identified in our previous study [4] was involved in the biogenesis of 5*′-tRF-GlyGCC* both in vitro and in vivo. The expression of ALKBH3 can positively regulate the generation of 5*′-tRF-GlyGCC* in blood cells. Further, blood cells co-cultured with CRC cells or mice xenografted with CRC tumors can increase the biogenesis of 5*′-tRF-GlyGCC* via an ALKBH3-dependent manner. ALKBH3 has been suggested to function as a DNA-repair protein to protect genomic integrity [5, 39]. Our previous study has revealed that it can specifically demethylate m^1^A and m^3^C of tRNA to induce the generation of tDRs [4]. In addition, ALKBH3 is highly expressed in various cancers [29, 32] to trigger the cancer progression via induction of tDRs [4]. Also, ALKBH3 is suggested to be beneficial to the growth and progression of CRC cells [20]. The role of tDRs including 5*′-tRF-GlyGCC* in the promotion effects of ALKBH3 on cancer progression needs further study.

## Conclusions

We showed that the profile and abundance of tDRs in the plasma of CRC patients and highlighted that *5′-tRF-GlyGCC* is a promising biomarker for CRC diagnosis. Moreover, we found high levels of *5′-tRF-GlyGCC* in CRC might be due to the upregulation of tRNA demethylases ALKBH3 by analyzing data from cellular, tumor bearing mice. It should be noted that the composition of tDRs might be different in different tumor models; the role of *5′-tRF-GlyGCC* as a biomarker and its carcinogenesis in other types of cancer need further investigation.

## Supplementary Information

**Additional file 1: Fig. S1.** The standard curve of *5′-tRF-GlyGCC* quantification. **Fig. S2.** ALKBH3 is involved in the biogenesis of *5′-tRF-GlyGCC*. **Fig. S3.** The nude mice (A) and BALB/c (B) mice bearing CRC cells xenografted tumor. **Table S1.** Background information of the Small RNA sequencing samples. **Table S2.** Background demographic of the study cohorts. **Table S4.** Relationship between the levels of *5′-tRF-GlyGCC* and the clinicopathological variables in CRC patients. **Table S5.** Clinical diagnosis utility about various marker alone and their combination effects for CRC diagnosis.

**Additional file 2: Supplemental Table S3.** The detailed reads data of tDRs in plasma of CRC and HCs.

## Data Availability

All data generated or analyzed during this study are included in this published article and its Additional files.

## Abbreviations

ALKBH3: AlkB homolog 3
AUC: Area under the curve
CA: Carbohydrate antigen
CEA: Carcinoembryonic antigen
CRC: Colorectal cancer
EDTA: Ethylene diamine tetraacetic acid
FBS: Fetal bovine serum
FIT: Fecal immunochemical test
GAPDH: Glyceraldehyde-3-phosphate dehydrogenase
HC: Healthy controls
m^1^A: 1-Methyladenosine
m^1^G: 1-Methylguanosine
m^2,2^G: 2,2,-Dimethylguanosine
m^3^C: 3-Methylcytidine
OB: Occult blood
PP: Polypropylene
qRT-PCR: Quantitative real-time PCR
ROC: The receiver-operating characteristic
SD: Standard deviation
smRNAs: Small RNAs
tDRs: tRNA-derived small RNAs
tRFs: tRNA-derived small RNA fragments
tRNA: Transfer RNA
