# Supplementary material for: 5′-tRF-GlyGCC: a tRNA-derived small RNA as a novel biomarker for colorectal cancer diagnosis
Source: Genome Med. 2021 Feb 9;13:20. doi: 10.1186/s13073-021-00833-x (PMC7874477; doi:10.1186/s13073-021-00833-x)
Supplement: Supplementary file 1 — Additional file 1: Fig. S1. The standard curve of 5′-tRF-GlyGCC quantification. Fig. S2. ALKBH3 is involved in the biogenesis of 5′-tRF-GlyGCC. Fig. S3. The nude mice (A) and BALB/c (B) mice bearing CRC cells xenografted tumor. Table S1. Background information of the Small RNA sequencing samples. Table S2. Background demographic of the study cohorts. Table S4. Relationship between the levels of 5′-tRF-GlyGCC and the clinicopathological variables in CRC patients. Table S5. Clinical diagnosis utility about various marker alone and their combination effects for CRC diagnosis. [file 13073_2021_833_MOESM1_ESM.docx]

**Supplementary data for**

**5'-tRF-GlyGCC: A tRNA-derived small RNA as a novel biomarker for colorectal cancer diagnosis**

**Wu et al**

**Fig. S1. The standard curve of** ***5'-tRF-GlyGCC* quantification**


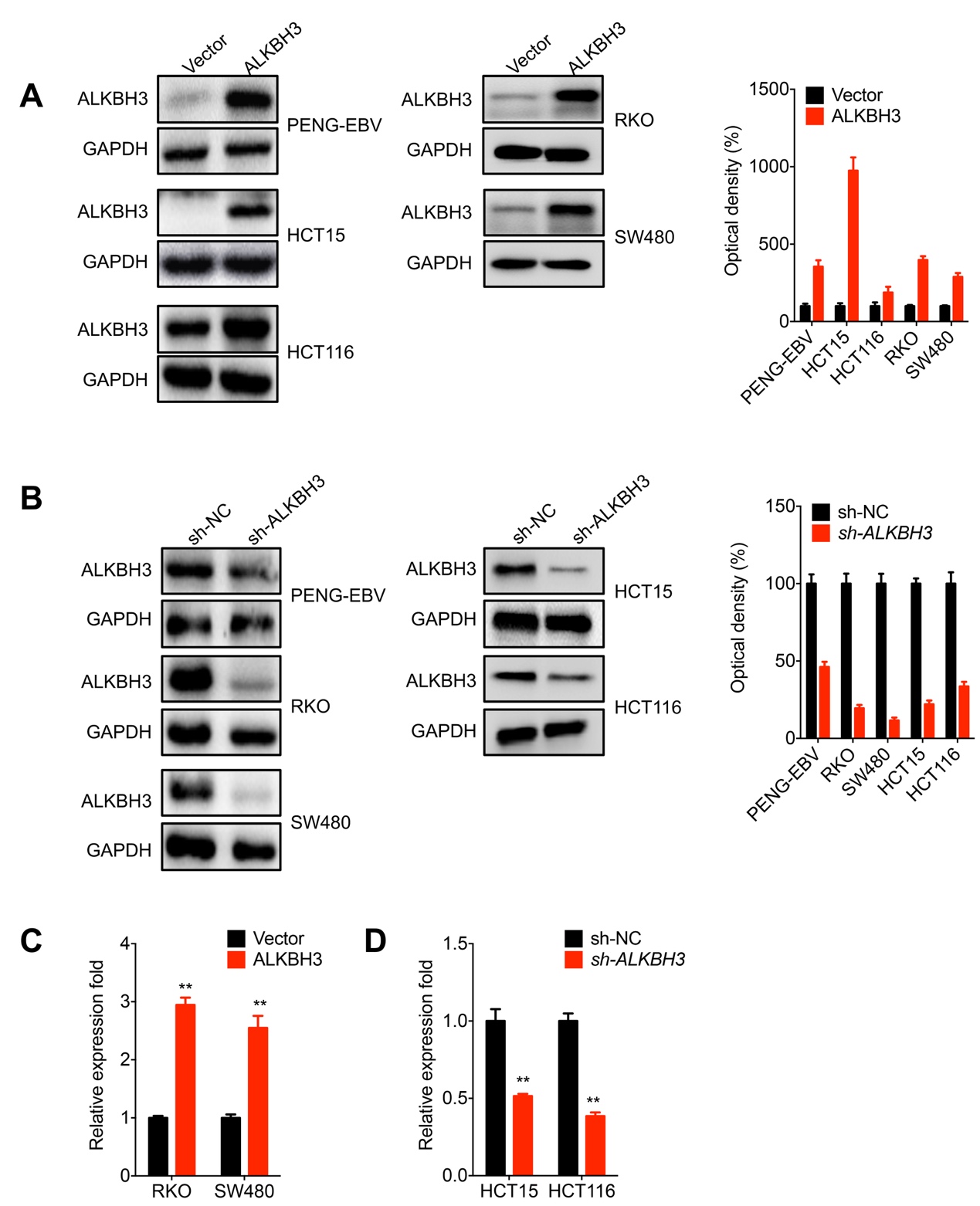


**Fig. S2. ALKBH3 is involved in the biogenesis of *5'-tRF-GlyGCC***

(A) Cells were transfected with the vector control or PPB/ALKBH3 for 48 h, and the expression of ALKBH3 was measured and quantitatively analyzed;

(B) Cells were transfected with the sh-NC control or sh-ALKBH3 for 48 h, and the expression of ALKBH3 was measured and quantitatively analyzed;

(C) Cells were transfected with the vector control or PPB/ALKBH3 for 48 h, and the expression of 5'-tRF-GlyGCC were measured by qRT-PCR;

(D) Cells were transfected with the sh-NC control or sh-ALKBH3 for 48 h, then the expression of 5'-tRF-GlyGCC were measured by qRT-PCR.

Data are presented as the mean ± SD from three independent experiments. ** *p*<0.01 compared with control.

**Fig. S3.** **The nude mice (A) and Balb/c (B) mice bearing CRC cells xenografted tumor**

**Table S1. Background information of the Small RNA sequencing samples**

| **Sample** | **Gender** | **Age (y)** |
| --- | --- | --- |
| HC1 | Female | 29 |
| HC2 | Male | 24 |
| HC3 | Male | 25 |
| CRC1 | Male | 69 |
| CRC2 | Male | 63 |
| CRC3 | Female | 79 |

**Table S2. Background demographic of the study cohorts.**

| **Parameter** | **HC** | **CRC** | **Total** |
| --- | --- | --- | --- |
| **N** | 90 | 105 | 195 |
| **Age** |  |  |  |
| Mean ± SD | 52.2±10.5 | 58.7±11.9 | 54.5±12.2 |
| Range | 30-80 | 33-84 | 30-84 |
| **Gender** |  |  |  |
| Male | 54  (60.0%) | 63  (60.0%) | 117  (60.0%) |
| Female | 36  (40.0%) | 42  (40.0%) | 78  (40.0%) |

**Table S4. Relationship between the levels of *5’-tRF-GlyGCC* and the clinicopathological variables in CRC patients.**

| **Variables** | **cases** | ***5’-tRF-GlyGCC*** | ***p* value** |
| --- | --- | --- | --- |
|  | **(n)** | **(ng/ml, mean**±**SD)** |  |
| **Age (y)** |  |  |  |
| <60 | 53 | 7.983±1.925 | 0.7262 |
| ≥60 | 52 | 7.135±1.447 |  |
| **Gender** |  |  | 0.8221 |
| Male | 63 | 7.785±1.772 |  |
| Female | 42 | 7.230±1.427 |  |
| **Metastasis** |  |  | 0.0241 |
| No | 62 | 4.807±0.6135 |  |
| Yes | 30 | 9.331±2.520 |  |
| **Location** |  |  | 0.7428 |
| Colon | 61 | 7.128±1.330 |  |
| Rectal | 43 | 7.940±2.256 |  |
| **Stage** |  |  | *VS* I |
| I | 11 | 4.597±1.633 |  |
| II | 34 | 7.991±2.598 | 0.4732 |
| III | 32 | 5.453±1.449 | 0.7491 |
| IV | 25 | 9.610±2.550 | 0.2208  *VS* HC |
| HC | 90 | 1.526±0.1144 |  |
| I | 11 | 4.597±1.633 | <0.0001 |
| II | 34 | 7.991±2.598 | <0.0001 |
| III | 32 | 5.453±1.449 | <0.0001 |
| IV | 25 | 9.610±2.550 | <0.0001 |
| **Ki67** |  |  | 0.5852 |
| <50 | 34 | 5.227±0.8429 |  |
| ≥50 | 38 | 6.648±2.330 |  |
| **CEA (ng/ml)** |  |  | 0.0318 |
| <5 | 47 | 4.570±0.6427 |  |
| ≥5 | 54 | 9.698±2.123 |  |
| **CA199 (IU/ml)** |  |  | 0.0420 |
| <37 | 74 | 5.591±0.8838 |  |
| ≥37 | 27 | 9.951±2.546 |  |
| **CA125 (IU/ml)** |  |  | 0.4523 |
| <35 | 92 | 7.169±1.284 |  |
| ≥35 | 14 | 9.828±3.252 |  |
| **KRAS2** |  |  | 0.7648 |
| WT | 36 | 6.683±2.433 |  |
| Mut | 23 | 5.725±1.142 |  |

**Table S5. Clinical diagnosis utility about various marker alone and their combination effects for CRC diagnosis.**

|  | **HC vs CRC** | | | | |
| --- | --- | --- | --- | --- | --- |
| **Marker** | **AUC** | **Sensitivity%** | **Specificity%** | ***p* value** | **95% CI** |
| ***5´-tRF-GlyGCC*** | 0.882 | 85.71 | 72.22 | <0.0001 | 0.83-0.92 |
| **CA199** | 0.557 | 32.38 | 96.47 | 0.1780 | 0.48-0.63 |
| **CEA** | 0.762 | 68.35 | 74.68 | <0.0001 | 0.69-0.83 |
| ***5´-tRF-GlyGCC* + CA199** | 0.910 | 82.86 | 80.00 | <0.0001 | 0.86-0.95 |
| ***5´-tRF-GlyGCC* +CEA** | 0.921 | 84.81 | 81.01 | <0.0001 | 0.85-0.95 |
| ***5´-tRF-GlyGCC* +CEA+CA199** | 0.926 | 86.08 | 84.00 | <0.0001 | 0.87-0.96 |
